# *Escherichia coli* and Sudden Infant Death Syndrome

**DOI:** 10.3389/fimmu.2015.00343

**Published:** 2015-07-03

**Authors:** Karl A. Bettelheim, Paul N. Goldwater

**Affiliations:** ^1^Discipline of Paediatrics, School of Paediatrics and Reproductive Health, University of Adelaide, North Adelaide, SA, Australia

**Keywords:** sudden infant death syndrome, SIDS, *Escherichia coli*

## Abstract

This review examines the association of strains of *Escherichia coli* with sudden infant death syndrome (SIDS) and the possible role these bacteria play in this enigmatic condition. The review addresses evidence for *E. coli* in SIDS infants, potential sources of *E. coli* in the environment, colonization by commensal and pathogenic strains, the variety of currently accepted pathotypes, and how these pathotypes could compromise intestinal integrity and induce inflammation. Both intestinal and extraintestinal pathotypes are compared in relation to the apparent liability in which virulence traits can be gained or lost by strains of *E. coli*. The way in which *E. coli* infections fit with current views on infant sleeping position and other SIDS risk factors is highlighted.

## Commensal and Pathogenic *Escherichia coli*

While *Escherichia coli* and “coliform” bacteria are considered part of the normal microbiome of the human intestinal tract, they have been identified in studies of infants who died suddenly and unexpectedly, infants classified either as sudden infant death syndrome (SIDS) and more recently sudden unexpected death in infancy (SUDI) (1–3). When confined to the gut, these bacteria are generally considered harmless; however, a number of pathotypes of *E. coli* can cause disease in humans and animals. The pathotypes are characterized by their ability to produce certain virulence factors, toxins and adhesins. They include enteropathogenic *E. coli* (EPEC), enterotoxigenic *E. coli* (ETEC), enteroaggregative *E. coli* (EAgEC), Shigatoxigenic *E. coli* (STEC) [verotoxigenic *E. coli* (VTEC)], enteroinvasive *E. coli* (EIEC), and diffuse adherent *E. coli* (DAEC). Their characteristics will be considered in more detail below.

## All *E. coli* are Not the Same

Until 1940s, pathogenic *E. coli* were not identified; they could not be distinguished by techniques available from non-pathogenic commensal *E. coli*. The pioneering work of Kauffmann in developing a serotyping scheme enabled strains belonging to certain O:H serotypes to be linked to certain of the pathotypes. For many years, EPEC was only recognized by serotypes until specific virulence factors were identified.

Mechanisms by which the pathogenic *E. coli* gain access to and remain within a susceptible host were unknown but considered likely to be similar to that of commensal *E. coli* over extended periods of co-evolution. Studies on commensal *E. coli* in 1970s provided a basis for understanding the colonization of the human body by *E. coli*.

### Commensal strains

In the first of these studies (4), the complete feces from 9 healthy adults were examined with 10 sites on each specimen assessed and at least 10 *E. coli*-like colonies collected from each site. These revealed a great diversity of types, although in all stools except 1 a single predominant type was present at all 10 sites. This indicated that generally when an *E. coli* isolate is selected from a fecal specimen, it is likely to be the predominant type, but minor types might be missed.

An extensive study on neonates’ acquisition of *E. coli* (5) found “*E. coli* are present in the vagina of women and that the acquisition of these *E. coli* by babies is related to the length of time that the birth takes. It was also noted that there is a relationship between the *E. coli* found in the feces of the mothers, the mucus swallowed by the babies at birth, and subsequently in the feces of the babies’. While this might be how infants acquire their *E. coli* and other microflora, it was noted that “Caesarian section babies are generally not likely to become colonized by their mothers’ fecal *E. coli*.” They were, however, colonized as rapidly as vaginally delivered babies. These studies showed that the babies’ gut was colonized early and these colonized babies became the foci for spread of *E. coli* to other babies. Environmental studies indicated that “contaminated hands and uniforms of the nursing staff may be the main vector for transmitting *E. coli*.” It was also noted that these *E. coli* acquired or lost certain traits during their spread including antibiotic resistance, serological variation, and even biochemical characteristics (6). Spread of *E. coli* was to a maximum of four other infants; Caesarian babies were particularly prone to colonization by these types of *E. coli*.

### Pathotypes

Studies on commensal *E. coli* summarized above were in part inspired by two earlier studies. The first was initiated as a result of a survey on *E. coli* urinary-tract infection (UTI) among patients who had been admitted to a female general medical ward over a period of 2 years (7). The use of an extensive set of *E. coli* typing antisera enabled a more complete identification and analysis. It was noted that the O types responsible for most of the infections belonged to relatively few common types.

Patients with diabetes, malignant disorders, rheumatoid arthritis, and hypertension had a much higher incidence of *E. coli* UTI than the rest of the ward population. In addition, there were small epidemics of UTI due to specific *E. coli* O types in the ward. These studies showed that none of these was acquired by catheterization or instrumentation. The evidence accorded with an oral transmission of type-specific *E. coli* from the intestinal reservoirs of human carriers.

Among 20 patients (8) who had previously been free of UTI and who developed infection, 13 developed these infections within 12 days of gynecological operations accompanied by insertion of a self-retaining catheter. The infections were caused by a type, which had been present before the operation in the rectum, vagina, or both.

These studies indicate that commensal *E. coli* can be transmitted among infants and individuals who are susceptible can acquire such *E. coli*, observations that could be relevant to susceptibility of both neonates (especially premature) and women who developed UTI.

Investigations carried under the sponsorship of the military authorities examined the cause of travelers’ diarrhea, which affects individuals newly arrived in a foreign country, usually within 14 days of arrival (9). A group of 540 British soldiers traveled by air from England to Aden; of these 38 had an episode of diarrhea during their first 14 days after arrival. Fecal specimens from 35 were investigated and 2 yielded strains of *Salmonella* spp., no known pathogens were isolated from the remaining 33. A new serotype of *E. coli* (O148.H28) was isolated in the acute phase from 19 of these cases. In the remaining 14 (40%) cases, a number of different *E. coli* serotypes were isolated. These included serotypes associated with infantile diarrhea and not related to travelers’ diarrhea. The peak of the isolations of *E. coli* O148:H28 corresponded with the peak incidence of travelers’ diarrhea. This serotype was never isolated from a healthy subject. A year later, in a laboratory in England, a technician working with this serotype developed a severe episode of diarrhea and *E. coli* O148:H28 was recovered in pure culture from his stools. The same serotype as well as O6:H16 were subsequently isolated from American soldiers in Vietnam (10).

Since these early discoveries at least 78 detectable O antigens and 34 H antigens associated with ETEC have been described. Among the most common serotypes, which produce either the so-called heat stable enterotoxin (ST), heat labile enterotoxin (LT), or both are O6:H16 (LT/ST), O8:H9 (ST only), O25:NM (LT only), O78:H12 (ST only), O148:H28 (ST only), and O153:H45 (ST only). Their prevalence and phenotypes [based on the toxins they carry or the colonization factors (CFs) they express] can vary depending on the location. Many of these serotypes were also commonly isolated from environmental sources and human cases and outbreaks around the world (11). These studies confirmed that *E. coli* can be true pathogens as well as commensal and they can be acquired and shed by humans.

In 1983, a mild outbreak of diarrhea occurred in the neonatal ward (12), in which the earlier studies on the spread of commensal *E. coli* had been carried out (5, 6). Despite introduction of strict control measures, the epidemic strain (O125:K70:H21) spread to 16 infants while the commensal strains spread maximally to 4 infants, similar to the spread observed in the previous study for which no specific containment had been undertaken. Commensal *E. coli* spread to other infants unimpeded and the outbreak strain spread far more than the commensals.

Initially, *EPEC* was identified on the basis that certain serotypes were regularly associated with infantile gastroenteritis, but the actual virulence factors were not known. Following discovery of the virulence factors, they were shown to be present not only in the currently isolated EPEC strains but also in original archival EPEC strains, isolated long before the description of these virulence factors (13) demonstrating that EPEC comprised a specific lineage of pathogenic *E. coli*.

Shigatoxigenic *E. coli* or *VTEC* are another pathotype first described in 1977 (14) in a study of the effects of culture supernatants from various *E. coli* on different cell lines including Vero cells (14). While a number of different serotypes were identified as being associated with these pathotypes (15) one serotype (O157:H7), first described in 1983 (16) has dominated the STEC/VTEC literature. It was soon realized that the Verotoxins were similar to the Shiga toxin associated with strains of *Shigella dysenteriae*, which had been described as long ago as 1903 (17, 18). Non-O157 STEC/VTEC was generally overlooked in the early years of STEC/VTEC research, but their importance as causes of sporadic and outbreak disease is now well established (15).

It has been shown over the years that various “mobile genetic elements,” such as transposons, insertion sequences, bacteriophages, and plasmids, can exist either integrated into the chromosome or through self-replication within the new host to provide new traits and fitness advantages. Most definable virulence factors found in pathogenic *E. coli* are derived from genetic mobile elements. For example, most of the genes for toxins and CFs required for the pathogenesis of ETEC are found almost exclusively on plasmids (11). These plasmids can move between strains supplying a variety of palettes of virulence factors. A recent example of this type of interaction was shown with the mixing of virulence factors, resulting in one of the most serious outbreaks caused by *E. coli*. This was the outbreak in 2011 (19) due to enteroaggregative *E. coli* O104:H4 strains, which were also able to produce Shiga toxins and which affected around 4000 individuals in Germany and many more in 16 other countries. Recently, it was shown that the enteroaggregative strain had acquired the bacteriophage coding for Shiga toxin 2a in cattle (20). Most *E. coli* infections are enteric. Extraintestinal infections, such as UTI and neonatal meningitis, probably, also have an enteric origin (7, 8, 21). This could have implications in regards to SIDS (*vide infra)*.

## *Escherichia coli* and SIDS

Sudden infant death syndrome remains a major cause of death of infants under 1 year of age. It was defined as long ago as 1969 (22), and its cause remains enigmatic. A large number of studies by both us and others have suggested that a bacterial infection may be a contributing or causal factor (23–30). *E. coli* is often identified in these infants. α-Hemloysin (HLyA)-producing serotypes of *E. coli* causing urinary tract and other extraintestinal infections are more commonly isolated from SIDS infants (29). These include O1:H^−^, O1:H5, O2:H1, O4:H5, O6:H1, O25:H1, and O75:H5 (31). A recent study (32) found that the α-HLyA of *E. coli* triggers intestinal inflammation in mouse models of inflammatory bowel disease by impairing the intestinal barrier thereby intensifying antigen uptake. α-HLyA-producing *E. coli* were more commonly isolated from the human mucosa in patients with active ulcerative colitis than in controls. α-hemolysin-producing *E. coli* needs to be considered in SIDS. The effects of HLyA and/or Ehly on the enterocyte and gut wall integrity could result in mucosal perturbation or damage promoting translocation of bacteria (*E. coli* or other gut microbiota) into the bloodstream either through induced inflammation or directly [see below and the accompanying article by Goldwater (33)].

The α-hemolysin is synthesized as a 1024-amino acid polypeptide, and then intracellularly activated by specific fatty acylation. A second activation step takes place in the extracellular medium through binding of Ca^2+^ ions (Ca^2+^ HlyA) (34). Recent studies have further shown that α-HLyA of *E. coli* interacts directly with cholesterol (35). These studies note that the insertion of α-HLyA is favored in cholesterol- and sphingomyelin-containing membranes. These studies, which demonstrated a clear interaction between α-HLyA and cholesterol, show this favors a conformational change of the α-HLyA that permits its correct insertion into the membrane to form membrane pores. Some strains of *E. coli* produce a different hemolysin from α-hemolysin, i.e., enterohemolysin (Ehx or Ehly), which is especially associated with STEC, although not all STEC produce it. There are enterohemolysin-producing *E. coli* that do not produce the Shiga toxins (36).

*Enterohemolysin* was shown to be a toxin and a member of the RTX (repeats-in-toxin) family of toxins. In its free form, it is lytic toward human endothelial and intestinal epithelial cells. It is also associated with outer membrane vesicles (OMV). The role of these OMVs was largely unknown (37). Their investigation (37) involved microscopic, biochemical, flow cytometry, and functional analyses of human brain microvascular endothelial cells (HBMEC) and Caco-2 to examine the role of OMV-associated toxins. These demonstrated that OMV-associated enterhemolysin does not lyse the target cells but triggers their apoptosis. Following internalization of the OMV-associated enterhemolysin by HBMEC and Caco-2 cells, a cascade ensues, which lead to apoptotic cell death as demonstrated by DNA fragmentation and chromatin condensation in the intoxicated cells. This ability of OMV-associated enterohemolysin to trigger the mitochondrial apoptotic pathway in human microvascular endothelial and intestinal cells is considered to be a newly recognized mechanism for a bacterial toxin to enter host cells in order to target mitochondria. As far as is known, the production of enterolysin by SIDS-associated *E. coli* has not been fully investigated; however, we have observed Ehly to be common in SIDS strains (unpublished). A rapid test for the production of enterohemolysin by *E. coli* is available (38). STEC has been identified in bovine feces and non-shiga-toxigenic *E. coli*, which belong to typical STEC serotypes; these carry most of the STEC virulence factors apart from being Shiga toxigenic in many cases also produce enterohemolysin (36). Such *E. coli* are known as atypical enteropathogenic *E. coli* (aEPEC) and have also been found to be present in patients with STEC infections and it appears that the acquisition or loss of the phage coding for the production of Shiga toxin in patients infected with STEC (39) is a not uncommon phenomenon. This was first described in 2005 (40).

It has been demonstrated (41) that certain ETEC that cause travelers’ diarrhea produce a protein, EatA, a plasmid-encoded serine protease, which degrades MUC2, which is a major protein present in the mucus layer of the small intestine. The removal of MUC2 accelerates the access of the ETEC enterotoxins to the enterocyte surface. While this has not been sought in SIDS-associated *E. coli*, the fact that some of these strains can produce this factor does not preclude others also producing this or similar factors. Other studies (42) show that another factor, YghJ, is highly conserved in ETEC and is a metalloprotease that degrades the major mucins, MUC2 and MUC3. This metalloprotease has also been found to be present in other *E. coli*, including the enteroaggregative *E. coli* O104:H4 and even strains of *Vibrio cholerae*. These have not yet been investigated in SIDS.

Of relevance is a study in baboons on acute respiratory distress syndrome (ARDS) (43), which mimics *E. coli* sepsis in humans. The baboon model displays the early inflammatory phase with extensive necrosis. If certain *E. coli* can cause ARDS, then it is possible that a similar mechanism occurs in SIDS given that the clinical and pathological findings in SIDS babies reflect a process consistent with sepsis and/or toxemic shock. This is supported by sterile site evidence of bacteremia (33, 44, 45) and the knowledge that bacteremia is a profound inducer of hypoxemia (thought to be involved in the final pathway). This model is supported by clinical evidence of fever [sweat-soaked bedding (46) and raised rectal temperature (47) and the pathological findings of intrathoracic petechiae, heavy wet lungs, and liquid heart blood and elevated fibrin degradation products (48, 49)], findings compatible with a process of bacterial sepsis. The physiological findings of hypoxemia and bradycardia and asystole observed in monitored babies dying of SIDS (50), again point to bacterial sepsis or toxemia as the final event in SIDS.

Unfortunately, systematic studies have not been performed where isolates of *E. coli* obtained from cases of SIDS and from healthy infants have been subjected to a full battery of all the possible toxins and virulence factors that are known to be produced by *E. coli*. Perhaps, such systematic studies will be undertaken in the future. Direct toxin identification studies might be possible in body fluids, such as urine, in which kidneys concentrate or filter circulating bacterial toxins from the bloodstream. Preliminary unpublished data indicate urine to be a useful fluid for toxin detection utilizing advanced proteomic techniques (Morris, personal communication). This work could provide the breakthrough that has been too long awaited by SIDS researchers.

## SIDS Risk Factors in Relation to Acquisition of *E. coli*

### Bed sharing

An association between bed sharing and SIDS has been noted (51, 52); however, why bed sharing occurred on the occasion of the last sleep has not always been clearly defined. A recent study (53) systematically reviewed the issue but only partially answered such questions as Was the child unsettled/unwell which led to the baby joining the parents in their bed? The review divided reasons for bed sharing into either “intentional” or “reactive;” “monitoring” the baby was given as a reason in 56.9% of situations (66.7% in Blacks) and “crying” in 32.4%. These results could indicate that SIDS babies were unwell on the last sleep. Clearly, further refinement in reasons for bed sharing and the interactions with other environmental factors is needed. Babies of families that regularly bedshare seem to be at low risk of SIDS (54). Also, the effect of parental drug/alcohol consumption has been taken to imply that this would lead to overlaying. An alternative hypothesis would be that drug-affected adults might not be alerted to an unwell/unsettled baby. An even more basic suggestion is that the infants acquire *E. coli* flora from close contact with parents or their bed linen.

While such questions remain unanswered, it is generally suggested that parents should share the room but not the bed with the infant (55, 56). An extensive study was undertaken in USA. to determine those trends and factors that might be involved with bed sharing over a period from 1993 to 2010 (57). This study, which was conducted through approximately 1000 telephone interviews annually, showed that there was a steady increase in bed sharing throughout the period of study. A further study suggested that breastfeeding might be affected by bed sharing (58) and *vice versa* (53). In another study (59), the authors identified as “three areas of concern” being (a) the baby sleeping in areas other than crib or bassinet, (b) bed sharing, and (c) non-supine sleeping.

### Mode of delivery

A number of environmental factors have been investigated with regard to acquisition of bacteria by neonates (4–6). Mode of delivery (Cesarean or vaginal delivery) being important. There are indications that bacterial characteristics also play an important role in dissemination, acquisition, and successful colonization (12).

### Used cot mattresses

The association between used cot mattresses and SIDS (60) was investigated by assessment of used polyurethane foam cot mattresses. The bacterial population density was greatly increased in used mattresses. *E. coli* was isolated from the dorso region of the mattresses, significantly so if the infant slept in the prone position, another significant risk factor for SIDS (61).

## SIDS Risk Factors in Relation to Acquisition of and Effects of *E. coli*

### Viral respiratory infections

Experimental studies have indicated that mild virus infection might potentiate the inflammatory response to bacterial toxins, particularly endotoxins. Moscovis et al. (this issue) (62) demonstrated in a model system that interferon-γ (IFN-γ) significantly enhanced pro-inflammatory response to endotoxin and significantly reduced the anti-inflammatory IL-10 response. The effects of IFN-γ on responses to the soluble *E. coli* toxins have not been examined.

### Environmental pollutants

A recent preliminary study (63) showed that the intestinal bacterial flora of neonates is influenced by house dust. Using high through-put sequence analysis of portions of the16S rRNA gene, it was found that despite significant differences between the dust and fecal microbiota as revealed by non-metric multidimensional scaling (NMDS) analysis, permutation analysis confirmed that 14 bacterial operational taxonomic units (OTUs) representing the classes Actinobacteria, Bacilli, Clostridia, and Gammaproteobacteria co-occurred at a significantly higher frequency in matched dust–stool pairs than in randomly permuted pairs, indicating an association between these dust and stool communities.

A study (64) of house dust and its relation to the skin surface swab samples of occupants in four homes were analyzed for their bacterial content using more accurate culture-independent methodology. Species-level operational taxonomic units (SLOTUs) were used to compare the results. Phylogenetic description of SLOTUs of the bacterial sequences was analysed from the different house dusts and skin surface swabs, which represented random samples of bacteria present in a given sample. The study (65) showed that the presence of bacterial endotoxins in house dust and investigated the role that they may play in the development of allergen sensitization. As LPS is also a powerful inducer of inflammatory responses, these findings require more investigation.

A preliminary investigation of the gut micro flora in SIDS compared the intestinal contents from 52 SIDS cases, with 102 fecal samples from age-matched live comparison infants (66). These were screened by PCR to target 16s RNA genes of *Clostridium innocuum*, *Clostridium perfringens*, *Clostridium difficile*, *Bacteroides thetaiotaomicron*, and *Staphylococcus aureus*. While *E. coli* were not included in these studies, the distribution of the test microorganisms can be used to extrapolate the possible roles of *E. coli*. The paper showed that the gut microbiome of SIDS babies differs from that of normal babies. The major finding was significantly more babies dying prone were colonized by *S. aureus* colonization than babies similarly colonized but dying in other positions (supine/side) (odds ratio = 20.25; CI = 3.60–134.92). This supports the hypothesis that prone sleep position increases the risk of such colonization by *S. aureus* and *E. coli* (61) or induction of temperature dependent toxins, such as the staphylococcal pyrogenic toxin (67), as the nasal temperature of infants in the prone position is significantly raised. There was a highly significant association between pronesleeping in both gut and a sterile site infection (OR = ∞; CI = 2.04–∞). There is a large body of evidence that bacterial infection plays a role in the events triggering SIDS. [Many of the pathology findings are compatible with a process of bacterial sepsis together with the physiological findings of hypoxemia and bradyasystole followed by gasping, (50) point to bacterial sepsis/toxemia as the final event in SIDS pathogenesis.]

## *E. coli* in SIDS and Inflammation

A history of diarrheal illness in the last week of life is a frequent finding in SIDS (46). Nelson et al. (54) supports the probability of the occurrence of gut inflammatory changes in SIDS. In general inflammation of the gut in SIDS has not been widely studied, but supportive evidence of compatible histological changes has been published (68) and examination of autopsy reports frequently shows hyperplasia of germinal centers and follicular hyperplasia of mesenteric lymph nodes.

A recent study (69) showed that infection by enteric pathogens, such as *Salmonella enterica* serovar Typhimurium (S. Tm), is antagonized by a highly complex intestinal microbiome, i.e., colonization resistance. Disruption of colonization resistance by such conditions as antibiotic therapy, a germfree state or an immature microbiome of low complexity results in increased susceptibility to oral infection with pathogens of the *Enterobacteriaceae* family (70–72). Pathogen (e.g., S. Tm)-induced gut inflammation promotes parallel blooms of S. Tm and host commensal *E. coli*. These blooms out-compete residual microbiota with *E. coli* and other *Enterobacteriaceae* taking advantage of the inflammatory conditions. The bacteria compete successfully for resources by directly antagonizing competitors by producing antimicrobials including bacteriocins. The bacteriocins produced by *Enterobacteriaceae* are colicins. Blooms of commensal and pathogenic *Enterobacteriaceae* exploit the abundant conditions provided by the inflamed gut (73–75). In man, up to 15 different *E. coli* strains can be detected in the gut microbiome (72). The success of the inflammation-induced enterobacterial bloom has been attributed to colicins, such as ColIb (cib), which provides effective defense against competing bacteria. Expression of cib and CirA (the cognate outer membrane receptor of cib on colicin-sensitive *E. coli*), is regulated by iron limitation. In the context of SIDS, and on the basis of epidemiological evidence of diarrheal symptoms in the days preceding death, and the sterile site data (33, 44, 45) it is plausible that invoked inflammation of the gut could lead to translocation of bacteria from the gut lumen into the portal and systemic blood stream and thence provide the stimulus for a lethal cytokine storm. It is clear that detailed analysis of the gut in SIDS and the microbiome within are neglected areas of research. In time, this approach might provide important clues to the SIDS enigma. If the studies by Gómez-Moreno et al. (76) are shown to be universally applicable then testing fecal specimens for the several genes of bacterial origin, which had been previously associated with inflammation may well be seen as risk factors for SIDS and treatment to remove these bacteria may well be achievable. Until such studies (76) are universally undertaken as part of the forensic investigation into the sudden death of an infant, full conclusions cannot be drawn.

## Conflict of Interest Statement

The authors declare that the research was conducted in the absence of any commercial or financial relationships that could be construed as a potential conflict of interest.
